# Cost-Effectiveness Analysis of Local Ablation and Surgery for Liver Metastases of Oligometastatic Colorectal Cancer

**DOI:** 10.3390/cancers13071507

**Published:** 2021-03-25

**Authors:** Matthias F. Froelich, Moritz L. Schnitzer, Nils Rathmann, Fabian Tollens, Marcus Unterrainer, Shereen Rennebaum, Max Seidensticker, Jens Ricke, Johannes Rübenthaler, Wolfgang G. Kunz

**Affiliations:** 1 Department of Radiology and Nuclear Medicine, University Medical Center Mannheim, Theodor-Kutzer-Ufer 1-3, 68167 Mannheim, Germany; Matthias.Froelich@medma.uni-heidelberg.de (M.F.F.); Nils.Rathmann@medma.uni-heidelberg.de (N.R.); Fabian.Tollens@medma.uni-heidelberg.de (F.T.); Shereen.Rennebaum@umm.de (S.R.); 2 Department of Radiology, University Hospital, LMU Munich, Marchioninistr. 15, 81377 Munich, Germany; moritz.schnitzer14@gmail.com (M.L.S.); Marcus.Unterrainer@med.uni-muenchen.de (M.U.); max.seidensticker@med.uni-muenchen.de (M.S.); direktion.radiologie@med.uni-muenchen.de (J.R.); Johannes.ruebenthaler@med.uni-muenchen.de (J.R.)

**Keywords:** cost-effectiveness, ablation, omCRC

## Abstract

**Simple Summary:**

Colorectal cancer is among the most prevalent cancers worldwide, with every second patient developing liver metastasis during their illness. For treatment of oligometastatic patients, there are several alternatives such as surgery, microwave ablation (MWA) and radiofrequency ablation (RFA). Our aim was to evaluate the cost-effectiveness for each strategy, accounting for short- and long-term costs and efficacy. The analysis demonstrated that MWA and surgery offer a comparable efficacy. MWA was the most cost-effective strategy in intermediate resource settings, and can be considered a cost-effective alternative to surgery in high resource settings.

**Abstract:**

Background: Colorectal cancer is among the most prevalent cancer entities worldwide, with every second patient developing liver metastases during their illness. For local treatment of liver metastases, a surgical approach as well as ablative treatment options, such as microwave ablation (MWA) and radiofrequency ablation (RFA), are available. The aim of this study is to evaluate the cost-effectiveness of RFA, MWA and surgery in the treatment of liver metastases of oligometastatic colorectal cancer (omCRC) that are amenable for all investigated treatment modalities. Methods: A decision analysis based on a Markov model assessed lifetime costs and quality-adjusted life years (QALY) related to the treatment strategies RFA, MWA and surgical resection. Input parameters were based on the best available and most recent evidence. Probabilistic sensitivity analyses (PSA) were performed with Monte Carlo simulations to evaluate model robustness. The percentage of cost-effective iterations was determined for different willingness-to-pay (WTP) thresholds. Results: The base-case analysis showed that surgery led to higher long-term costs compared to RFA and MWA (USD 41,848 vs. USD 36,937 vs. USD 35,234), while providing better long-term outcomes than RFA, yet slightly lower than MWA (6.80 vs. 6.30 vs. 6.95 QALYs for surgery, RFA and MWA, respectively). In PSA, MWA was the most cost-effective strategy for all WTP thresholds below USD 80,000 per QALY. Conclusions: In omCRC patients with liver metastases, MWA and surgery are estimated to provide comparable efficacy. MWA was identified as the most cost-effective strategy in intermediate resource settings and should be considered as an alternative to surgery in high resource settings.

## 1. Introduction

Colorectal cancer is among the most relevant cancer entities, in regard to both cancer-related mortality and morbidity worldwide [1]. About 50% of patients will experience metastatic disease (mCRC)—most commonly in the liver [2]. Oligometastatic colorectal cancer (omCRC) is a state with 3–5 liver metastases arising from a colorectal cancer through the portal venous system [3]. For classification of metastatic disease, the concept of oligometastatic cancer as an intermediate state between localized and disseminated disease has been proposed [4,5]. In this context, local ablative treatments of patients with oligometastatic disease gain further importance as potential alternatives to surgical approaches [6]. In the past, surgery was seen as the gold standard in treatment of liver metastases, as it was regarded as the only approach to achieve a cure. Over the last few years, local ablative therapies, such as radiofrequency ablation (RFA), microwave ablation (MWA) or cryoablation, have complemented the treatment of oligometastatic liver diseases, especially for patient groups that are not suitable for surgery, e.g., older patients with comorbidities or metastases near important vessels [7]. In subgroups, local treatment of oligometastatic disease can even be curative [8]. Therefore, ablative treatment is recommended as a potential therapeutic modality in current guidelines [9]. Apart from thermal ablation techniques, other interventional treatment modalities, such as brachytherapy and stereotactic ablative radiotherapy, gain importance [10]. Further, for patients unable to undergo surgical treatment or after chemotherapy failure, transarterial chemoembolization (TACE) can be a valuable option for treating liver metastases [11]. This study, however, is focused on ablative and surgical treatment options.

The performance of RFA and MWA have been studied comprehensively in comparison to surgery [12], and these studies focus on metastatic disease that allows for the application of either treatment modality, e.g., based on tumor location and size. While local ablative treatments are associated with a reduced length of hospital stay [13] and lower initial costs, surgery has been advocated as the reference standard for complete resection. As a result, an assessment of treatment modalities for oligometastatic colorectal cancer (omCRC) with liver metastases should take both efficacy and short- and long-term costs into account.

Therefore, the aim of this study is to analyze the comparative cost-effectiveness of surgery, RFA and MWA in patients amenable to all investigated treatment modalities for the management of liver metastases in omCRC. 

## 2. Results

### 2.1. Cost-Effectiveness Analysis

In the base-case scenario (WTP USD 100,000 per QALY), the strategies surgery, RFA and MWA resulted in lifetime costs of USD 41,848; 36,937 and 35,234 with an effectiveness of 6.80, 6.30 and 6.95 QALYs. As a result, surgery and RFA were dominated by MWA in the base-case scenario. As an overview for the Markov model, the stage-dependent states after successful treatment are shown in Supplemental Figure S1.

### 2.2. Sensitivity Analysis 

**Figure 1:**
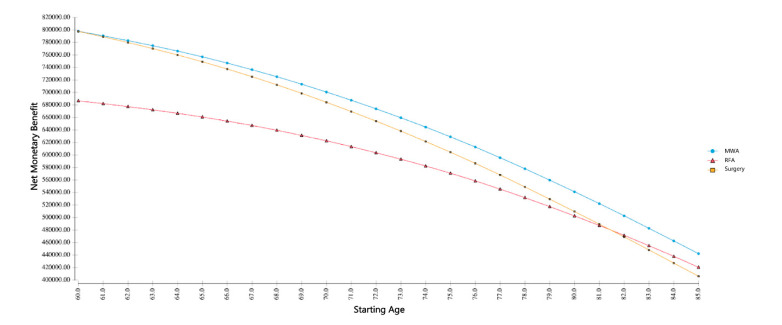
Age-dependent net monetary benefits based on a WTP of USD 100,000 per QALY. For a patient aged under 60 years, surgery yields the highest net monetary benefits; for other patients, microwave ablation results in the highest net monetary benefit.

To investigate the comparative cost-effectiveness for several thresholds of patient age, a corresponding cost-effectiveness analysis was performed (Figure 1). This resulted in MWA being a suitable option for a broad range of patients, with a tendency towards surgery for younger and towards RFA for older patients. Based on a WTP of USD 100,000 per QALY, MWA yielded the highest net monetary benefit (NMB) in a starting age ranging from 60 to 85 years. In particular, the NMB ranged from USD 800,000 for MWA and surgery for a patient aged 60 years, to consistently more than USD 400,000 for all modalities in the case of a patient aged 85 years. The NMB is defined as the difference of the product of effectiveness and WTP and the actual costs of a treatment [14,15].

**Figure 2:**
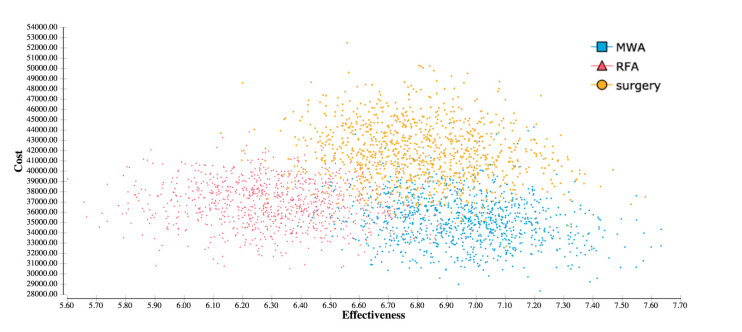
Cost-effectiveness scatterplot based on 30,000 iterations shows a higher effectiveness of MWA and surgery when compared to RFA. Additionally, MWA results in average lower costs than surgery.

**Figure 3:**
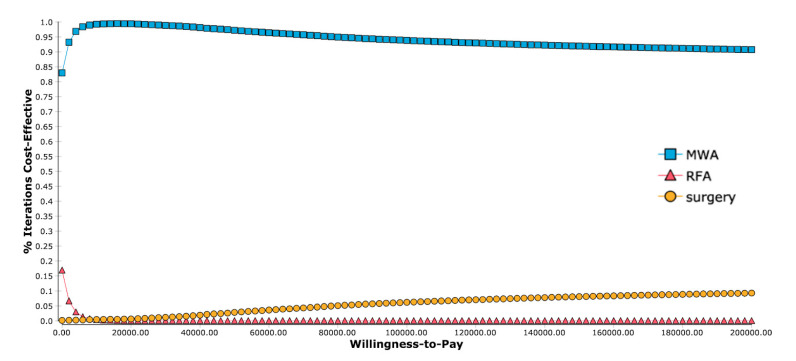
Acceptability curves for willingness-to-pay levels. In the range investigated, MWA is the cost-effective strategy in the majority of iterations.

To further investigate the influence of all input variables, a probabilistic sensitivity analysis was performed. This analysis showed results in favor of MWA, with an overall higher effectiveness than RFA and lower overall costs than surgery (Figure 2). A WTP-dependent analysis showed that for all WTP thresholds from USD 0 per QALY to USD 200,000 per QALY, MWA was a cost-effective strategy in more than 80% of iterations (Figure 3).

## 3. Discussion

In this model-based cost-effectiveness analysis comparing RFA, MWA and surgery in the setting of omCRC, surgery was compared to the two most common imaging-based ablation techniques for liver metastases. Based on the prerequisite that all treatment modalities are equally applicable, this analysis applies to patients that comply with the most important selection criteria, such as tumor location, size and number. In this patient scenario, our analysis showed that MWA is a cost-effective alternative for the treatment of omCRC liver metastases compared to RFA and surgery. MWA turned out to be the treatment option with the lowest overall cost and the highest cumulative cost-effectiveness for the patients, deeming it the dominant modality.

The cost-effectiveness of image-guided ablation techniques and surgery for treatment of liver metastases has already been raised in past studies. For instance, Gazelle et al., 2004 investigated the cost-effectiveness of percutaneous RFA compared to hepatic resection in mCRC, concluding that RFA may in fact be a cost-effective strategy, but the effectiveness of hepatic resection was superior in the majority of cases. It has to be emphasized that not only the technical quality of ablation treatment, but also the data availability, improved between the two analyses. By now, the complete ablation success of 93% with RFA is far superior to the reported success rates between 31.6 and 78.4%, depending on the size of the metastases in Gazelle et al., 2004, indicating that there might be a shift in cost-effectiveness due to improved performance of ablative methods or improved patient selection over time [16].

Additionally, Gazelle et al., 2003 showed in a study investigating the cost-effectiveness of hepatic metastasectomy in mCRC patients, that a more aggressive surgical approach entails a better and more cost-effective outcome compared to a less aggressive procedure [17]. Over time, the number of performed ablation techniques increased significantly. This development yields the necessity of an appropriate and cost-effective follow-up after the ablation of liver metastases. More recent economic analyses have analyzed the topic of imaging after ablation of liver metastases in mCRC. Schnitzer et al., 2020 examined the cost-effectiveness of follow-up after ablation of colorectal liver metastases by comparing contrast-enhanced CT and 18F-FDG PET/CT, showing that 18F-FDG PET/CT is the cost-effective choice for follow-up [7]. In addition, the cost-effectiveness of resection or ablation of other tumor entities has been analyzed. Cucchetti et al. aimed to identify the most economical treatment option for early hepatocellular carcinoma (HCC). RFA turned out to be the cost-effective approach in singular HCCs smaller than 2 cm and multiple HCCs smaller than 3 cm, whereas resection was more cost-effective for single HCCs measuring 3–5 cm [18]. Further, the ongoing COLLISION trial investigates the effectiveness of surgery and thermal ablation in small colorectal liver metastases (≤3 cm). The results of this trial will have an impact on the treatment strategies in the future and may, therefore, lead to a shift in cost-effectiveness of small colorectal liver metastases treatment [19].

Although our study showed an improved cost-effectiveness of MWA, the following limitations have to be taken into account. Our study may not be applicable as a general guideline for all patient cases, as the main question of our analysis is very specific and has an explicit focus on MWA, RFA and surgery in resectable colorectal liver metastasis that are amenable to all investigated treatment modalities. Every investigation results in different efficacies and may, therefore, result in deviations when considering several divergent input parameters. We tried to focus our analysis on the best input data available and applied them in our model. Nonetheless, we cannot guarantee that our study fits every scenario, but we consider the results of our model as a point of reference in the cost-effectiveness of colorectal liver metastases treatment. We were not able to consider different surgery techniques for the treatment of colorectal liver metastasis as there is a lack of adequate input data. Further, we assumed a second session treatment performance of 100% (Table 1). This approximation may deviate from clinical reality in some cases. This limitation results from the lack of data on second session treatment. Besides, a chemotherapeutic approach was excluded from the analysis. Ruers et al. showed that RFA in combination with systemic treatment is in fact a superior treatment option and provides better outcomes [20]. However, as this analysis only investigated the effectiveness of RFA and systemic therapy in unresectable liver metastases, the results are not applicable to our study which focuses only on resectable liver metastases. In general, the topic of unresectable liver metastases of colorectal cancer, and also conclusions concerning other oncologic modalities, have to be regarded as a subject of future studies. 

In general, every model has to rely on some simplifications and on certain modeling assumptions. The analysis strongly depends on the available input data. In our analysis, we aimed to depict the most realistic outcome possible based on the most appropriate and contemporary data. For some of our model input data, we needed to adapt to limited resources, which may influence the results. In particular, the data input based on Creasy et al. and Abdalla et al. analyzed survival and recurrence rates of surgery in patients with resectable colorectal liver metastases in general, and not specifically in patients with omCRC. As specific recurrence and survival values of omCRC surgery were not available, this limitation needs to be taken into account as a potential bias when interpreting the results. Further, the treatment success probabilities in this model may depend on experience and the setting of treatment. Therefore, the parameters may deviate between individual surgeons and interventional radiologists. Furthermore, efficacy parameters are subject to ongoing research and technical development. For example, additional transcatheter hepatic angiography (CTHA) is an emerging technique used for tumor visualization in ablative therapy in order to achieve better local tumor control and may result in better efficacies [21]. However, there are not enough adequate data available to include this technique in our analysis. Nevertheless, the analysis of an additional CTHA during ablation might be a promising topic for future studies. The presented modeling results may therefore only apply in settings which are represented realistically by the input parameters. In the case of a significant shift of input data values, the results of our analysis and therefore the cost-effectiveness results may change. 

The economic evaluation of treatment modalities of oligometastatic liver disease remains an important topic, as this patient collective will further grow due to demographic changes and enhanced survival due to improved medical treatment [22]. Therefore, not only the increasing number of necessary treatments and in course surging expenses, but, moreover, the fact that the resources of healthcare systems are limited, call out for a shift to a more value-based care, in order to provide the best care and treatment for all assured persons of a healthcare system. Hence, the American Society of Clinical Oncology (ASCO) defined clinical benefit, toxicity and cost as the three substantial columns of value-based healthcare in cancer treatment on which basis the treatment strategies should be evaluated [23]. Therefore, the purpose of a more value-based healthcare in cancer treatment and radiology is to optimize the clinical outcomes without increasing the costs. The subject of radiology takes an important place in this proposition as it should not be seen just as image acquisition, but more as an intersection that has an enormous impact on the further treatment of patients and the quantity of expenses needed for the treatment.

Although stereotactic ablative radiotherapy is an increasingly utilized strategy in patients with mCRC [24], this modality was not included in the model due to distinctly different mechanisms of action and lack of adequate head-to-head datasets. In addition, this cost-effectiveness analysis focuses on the situation of the US healthcare system and application to the setting of other healthcare systems should be performed with caution.

Aside from traditional criteria such as tumor size and location, the selection of the optimal treatment option also depends on patient age, as younger and less morbid patients are better suited for surgery. Older patients, often with comorbidities, are not fit enough for resection and, therefore, are better suited for minimally invasive treatment. To investigate this topic, a dedicated deterministic sensitivity analysis was performed. In the performed sensitivity analysis, the age-dependent net monetary benefit was calculated. It showed a lower net monetary benefit for RFA and, beginning with age 60, a higher net monetary benefit of MWA compared to surgery, showing the economic superiority of MWA, even with changing age ranges. At age 60, surgery and MWA yield equal net monetary benefit, suggesting that underneath this age, surgery might still be a cost-effective alternative. However, as the mean age of patients is 73 years, MWA may still be the most economical approach for the majority of patients.

In case patients are suitable for local ablative treatments, our results may lead to a reevaluation in choice of therapy towards MWA, as it offers excellent outcomes and is cost saving. Still, treatment decision making for patients largely depends on patients’ and treating physicians’ preferences. For example, tumor localization in certain liver segments may favor different techniques. Therefore, every treatment option should be chosen on a case-by-case basis in the setting of a multidisciplinary tumor board decision [9]. This analysis may give some guidance for decision makers on the payer level, as well as from a healthcare perspective. For the future, a cost-effectiveness analysis investigating RFA and MWA in combination with systemic therapy in unresectable liver metastasis should be performed in order to broaden the range of economic analyses in more advanced stages of mCRC.

Taken together, our current health economic evaluation extends the published literature and broadens the range of investigated modalities by including the modality of MWA. In conclusion, MWA can be regarded as a suitable alternative for surgery and RFA in hepatic omCRC from a current cost-effectiveness perspective.

## 4. Materials and Methods

### 4.1. Model Structure

**Figure 4:**
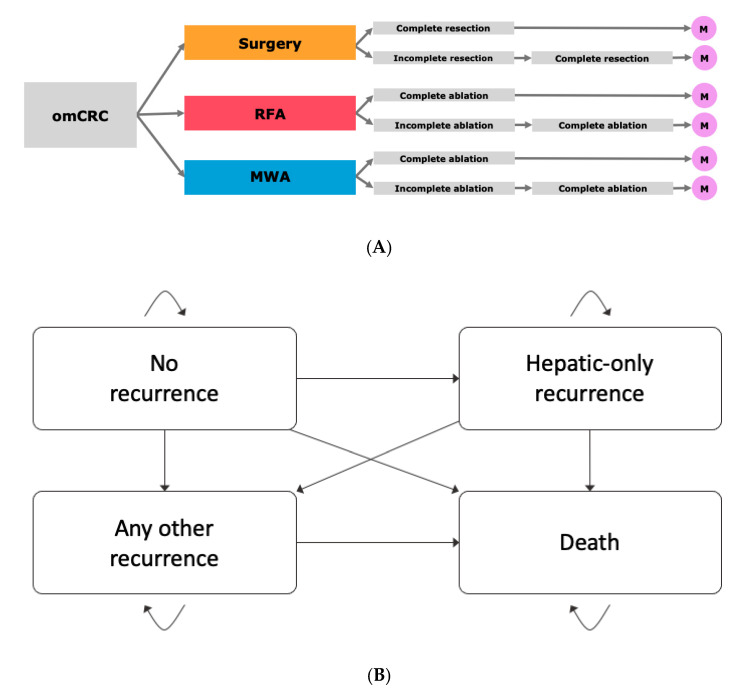
Model structure. (**A**) Overall model discriminating between the three treatment modalities surgery, microwave ablation (MWA) and radiofrequency ablation (RFA). (**B**) Markov Model for simulation of long-term outcomes containing the states “No recurrence”, “Hepatic-only recurrence”, “Any other recurrence” and “Death”.

To evaluate the cost-effectiveness of treatment strategies for liver metastases in the setting of omCRC, a decision model was developed (Figure 4A) using decision-analytic software (TreeAge Pro, version 19.1.1, Williamstown, MA, USA). At first, a short-run model, including costs and outcomes in the first 30 days was created. Additionally, long-term outcomes and costs were calculated in a long-run Markov model with a cycle length of one year. This model included the health states of no recurrence/hepatic recurrence only/any other recurrence/death (Figure 4B). A Markov model is defined as a stochastic simulation where the current status can be changed in the beginning of every cycle according to the predefined probabilities. In every stage of the model run, the patient can be allocated to a health state. Further, every health state can be allocated to presumed expenses and quality-of-life that are associated with this state.

### 4.2. Input Parameters

The age at ablation was set to 73 years in accordance with published patient collectives [25]. The maximum patient age was set to 100 years. Based on recommendations on cost-effectiveness analyses, the discount rate was set to 3% [26]. Additionally, willingness to pay (WTP) was set to USD 100,000 per quality-adjusted life year (QALY). All input parameters are summarized in Table 1 and Table 2.

**Table 1:** Input parameters for treatment performance, costs and quality of life for all treatment modalities.

Parameter	Surgery	Radiofrequency Ablation (RFA)	Microwave Ablation (MWA)
Treatment performance (% complete ablation/surgery)	99% Assumption, Gazelle et al., 2004 [16]	93% Shady W et al., 2018 [12]	97% Shady W et al., 2018 [12]
Treatment performance in second session	100% Assumption		
Acute costs for treatment	USD 2421 Medicare 2018	USD 1493 Medicare 2018	USD 1493 Medicare 2018
Average number of days in hospital	7NG KKC et al., 2017 [13]	4NG KKC et al., 2017 [13]	4 Assumption, NG KKC et al., 2017 [13]
Cost per day of hospital stay	USD 2,424 Henry J Kaiser Foundation 2017 [27]		
Calculated total treatment costs	USD 19,389	USD 11,189	USD 11,189
Quality of life 1 month after treatment	0.7 Gazelle et al., 2004 [16]	0.95 Gazelle et al., 2004 [16]	0.95 Gazelle et al., 2004 [16]

#### 4.2.1. Efficacy of Treatment Modalities

The ablation performance of RFA and MWA in the first session was set to 93% and 97%, based on literature comparing both modalities in one analysis [12]. The efficacy of surgery was set to 99% to account for its often-advocated position as the reference standard. In the case of an incomplete resection/ablation, a complete resection/ablation was assumed in a second approach for all treatment modalities. This approach has been utilized before for the modelling of ablation techniques [16].

#### 4.2.2. Costs and Utilities

Costs derived from Medicare in 2018 were included as initial treatment costs. To account for the different length of hospital stay for surgery and local ablation, length and daily cost of hospital stay were included in the analysis [13,27].

Utilities were collected as quality-adjusted life years (QALYs) based on the respective health state. For one month after treatment, a lower quality of life was used for surgery than for RFA and MWA [16]; however, the quality of life after more than one month was assumed to be solely based on recurrence status [31,32].

#### 4.2.3. Transition Probabilities

According to the Markov Model described above, probability of death, hepatic or non-hepatic recurrence were included in the analysis. US life tables were utilized for estimation of probability of death without recurrence. Further transition probabilities were collected from the literature [33,34,35,36,37].

#### 4.2.4. Cost-Effectiveness Analysis

According to the WTP—the assumed boundary of expenses for a patients’ medical benefit—and discount rate defined above, the expected QALYs and costs were calculated for a baseline scenario. Furthermore, incremental cost-effectiveness ratios (ICER) were estimated. An ICER reflects the economic value of a strategy. In this analysis, the results of the economic evaluation only refer to omCRC patients that are amenable to all investigated treatment modalities.

#### 4.2.5. Sensitivity Analysis

To analyze the robustness of the model and the effects of input parameters on the outcomes, a probabilistic sensitivity analysis was performed. For the latter, 30,000 Monte Carlo simulation iterations were applied. Based on the probabilistic analysis, acceptability curves were calculated. Further, to investigate the comparative cost-effectiveness for several thresholds of patient age, a corresponding cost-effectiveness analysis was performed.

## 5. Conclusions

In omCRC patients with liver metastases that are amenable to all investigated treatment modalities, MWA was identified as the most cost-effective strategy in intermediate resource settings and should be considered as an alternative to surgery in high resource settings. Nonetheless, MWA and surgery are estimated to provide comparable efficacy.

## Figures and Tables

**Table 2:** Additional input parameters for long term costs and outcomes.

Name	Estimate	Distribution	Source
Age at diagnosis	73		Kolligs FT et al., 2016 [25]
Discount rate	3.00%		Weinstein MC et al., 1996 [28]
**Costs (Long Term, Monthly)**			
Cost after treatment	USD 855	γ	Taplin SH et al., 1995/Assumption [29]
Cost hep./non-hep. recurrence	USD 3935	γ	Färkkilä N et al., 2015/Assumption [30]
**Utilities**			
QOL after >1 month: no recurrence	1	β	Fryback DG et al., 1993 [31]
QOL after >1 month: hepatic recurrence only	0.65	β	Kim et al., 2016 [32]
QOL after >1 month: any other recurrence	0.19	β	Kim et al., 2016 [32]
Death	0		
**Transition Probabilities**			
Probability of death without recurrence	US Life Tables	β	US Life Tables 2014
Probability of death with hepatic recurrence	12%	β	Masi G et al., 2009 [33]
Probability of death with non-hepatic recurrence	5%	β	Abdalla EK et al., 2004 [34]
1-year mortality after surgery	8.25%	β	Creasy et al., 2018 [35]
1-year mortality after RFA	6%	β	Bonne L et al., 2018 [36]
1-year mortality after MWA	5.50%	β	Bonne L et al., 2018 [36]
Probability of hepatic recurrence after surgery	2.50%	β	Abdalla EK et al., 2004 [34]
Probability of hepatic recurrence after RFA	7.70%	β	Correa-Gallego C et al., 2014 [37]
Probability of hepatic recurrence after MWA	4%	β	Correa-Gallego C et al., 2014 [37]
Probability of non-hepatic recurrence	5%	β	Abdalla EK et al., 2004 [34]

## Data Availability

No new data were created or analyzed in this study. Data sharing is not applicable to this article.

## Supplementary Materials

The following are available online at https://www.mdpi.com/2072-6694/13/7/1507/s1, Figure S1: Simulation of monthly Markov states after successful microwave ablation.
