# Supplementary material for: Cost-Effectiveness Analysis of Local Ablation and Surgery for Liver Metastases of Oligometastatic Colorectal Cancer
Source: Cancers (Basel). 2021 Mar 25;13(7):1507. doi: 10.3390/cancers13071507 (PMC8037107; doi:10.3390/cancers13071507)

cancers

Supplement

Figure S1: Simulation of monthly Markov states after successful microwave ablation

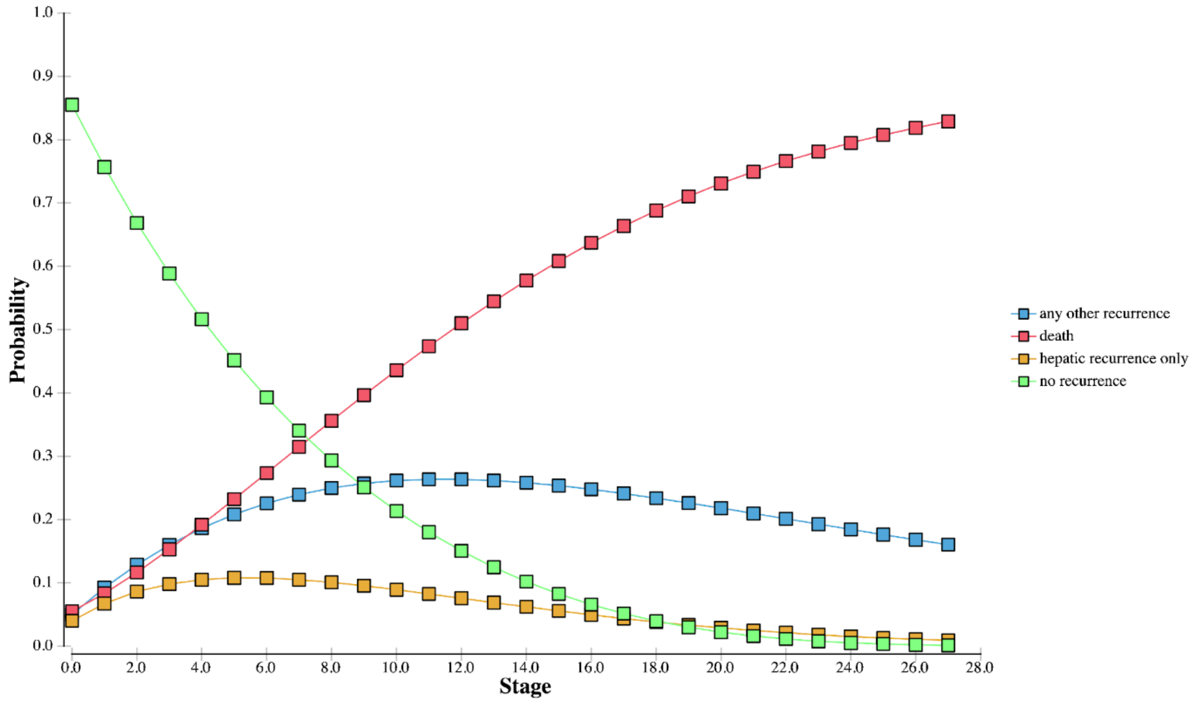

Supplement: Supplementary file 1 [file cancers-13-01507-s001.pdf]
